# The Effect of Superoxide Dismutase on Inhibition of Acute Kidney Injury Induced by Sepsis Based on Kidney Tissue Histology and Murine Sepsis Score

**DOI:** 10.1155/2021/1827296

**Published:** 2021-12-16

**Authors:** Jufitriani Ismy, Maimun Syukri, Dessy R. Emril, Nanan Sekarwana, Jufriady Ismy, Rachmad Suhanda

**Affiliations:** ^1^Graduate School of Mathematics and Applied Sciences, Universitas Syiah Kuala, Banda Aceh 23111, Indonesia; ^2^Department of Pediatrics, Faculty of Medicine, Universitas Syiah Kuala, General Hospital dr. Zainoel Abidin, Banda Aceh 23111, Indonesia; ^3^Department of Internal Medicine, Universitas Syiah Kuala, General Hospital dr. Zainoel Abidin, Banda Aceh 23111, Indonesia; ^4^Department of Neurology, Faculty of Medicine, Universitas Syiah Kuala, General Hospital dr. Zainoel Abidin, Banda Aceh 23111, Indonesia; ^5^Department of Pediatrics, Faculty of Medicine, Universitas Islam Bandung, Bandung 40161, Indonesia; ^6^Departement of Surgery, Urology Division Faculty of Medicine, Universitas Syiah Kuala, General Hospital dr. Zainoel Abidin, Banda Aceh 23111, Indonesia; ^7^Departement of Public Heath and Community Medicine, Universitas Syiah Kuala, Banda Aceh 23111, Indonesia

## Abstract

Sepsis is one of the leading causes contributing to the incidence of acute kidney injury (AKI). Oxidative stress can be used as the main approach against sepsis-induced AKI. One of the primary antioxidants that plays a role in warding off oxidative stress is superoxide dismutase (SOD). This research aimed to observe the effect of antioxidant SOD in inhibiting sepsis in AKI based on kidney tissue histopathology. The research method was an experimental laboratory with a post-test-only control group design. Twenty-five adult male rats aged 12–16 weeks, weighing between 200 and 250 g, were randomly divided into five groups: Group I, as a positive control, where rats were injected with lipopolysaccharides (LPS); Group II, as a negative control; Group III, as treatment 1, where rats were injected with LPS and administered orally with SOD (Glisodin®) 250 IU daily; Group IV, as treatment 2, where rats were injected with LPS and administered orally with SOD (Glisodin®) 500 IU daily; and Group V, as treatment 2, where rats were injected with LPS and administered orally with SOD (Glisodin®) 1000 IU daily. Rats were administered with SOD (Glisodin®) by oral gavage with a flexible feeding tube for 16 weeks, given once daily in the morning, and then injected with LPS of 10 mg/kg body weight. Glisodin SOD had a significant effect on murine sepsis score (MSS). MSS influenced the tubular injury score linearly. We conclude that the optimal dose of SOD at 1000 IU for inhibiting sepsis-induced AKI incidence is compared to SOD at a dose of 250 and 500 IU. The antioxidant effect of SOD can prevent sepsis-induced AKI with oxidative stress events.

## 1. Introduction

Sepsis is a fundamental problem in health care with a high mortality rate. The incidence of sepsis is reported to increase with increasing life expectancy associated with comorbid factors [1]. Sepsis does not always show the same symptoms in every patient. Based on sepsis characteristics, several parameters assess the sepsis disorder experienced by sepsis sufferers. A murine sepsis score (MSS) assessment is based on seven criteria: appearance, level of awareness, activity, response to stimuli, eyes, respiratory rate, and breathing quality [2].

Sepsis is one of the leading causes contributing to the incidence of acute kidney injury (AKI). Data on adults and children nationally in the United States showed that sepsis accounted for 26%–50% of AKI as a cause of AKI compared to 7%–10% of AKI due to primary kidney disease [3]. The high incidence of child AKI cases, namely, between 5% of inpatients and 30%–50% in intensive care units, proves that AKI is at a high alert level [4].

Several factors play a role in sepsis-induced AKI, including ischemic-reperfusion injury to the glomerulus, inflammation of the nephrons, hypoxia and oxidative stress, cytokines, and chemokines that cause tubular injury and apoptosis in mesenchymal cells [5–7]. Oxidative stress is considered as the main mechanism against sepsis-induced AKI [8].

Oxidative stress is a condition where there is an imbalance between oxidants and antioxidants in the body. One of the primary antioxidants responsible for counteracting oxidative stress is superoxide dismutase (SOD). Constantino et al. [9] stated that SOD is one of the natural antioxidant enzymes found in body cells due to several factors. As you grow older, this antioxidant activity can decrease so that antioxidants from outside the body are needed, one of which is Glisodin, a synthetic SOD. This study showed that the presence of SOD reduced levels of nitrotyrosine and proinflammatory cytokines that play a role in sepsis (*p* < 0.05).

Based on the above explanation, this study intended to determine the effect of the antioxidant SOD in inhibiting sepsis in AKI based on kidney tissue histopathology.

## 2. Materials and Methods

### 2.1. Reagents

Lipopolysaccharides (LPS, *Escherichia coli* O55 : B5) were purchased from Sigma-Aldrich (St. Louis, MO, USA). SOD extract was obtained as gliadin combined with standardized melon SOD (Glisodin®).

### 2.2. Animals and Treatments

Twenty-five adult male *Rattus norvegicus* rats (12–16 weeks), weighing 200–250 g, were obtained from the Experimental Animal Centre of Gadjah Mada University (Yogyakarta, Indonesia). The rats were acclimated for 1 week in the laboratory of Veterinary Medicine at Syiah Kuala University (Banda Aceh, Indonesia). The rats were allowed free access to water and food. Ethical clearance approval for using animals was obtained from the Veterinary Ethics Committee, Faculty of Veterinary Medicine, Syiah Kuala University (No. 20/KEPH/II/2019).

Rats were randomly divided into five groups: Group I, as a positive control, rats were injected with LPS; Group II, as a negative control; Group III, as treatment 1, rats were injected with LPS and administered orally with SOD (Glisodin®) 250 IU daily; Group IV, as treatment 2, rats were injected with LPS and administered orally with SOD (Glisodin®) 500 IU daily; and Group V, as treatment 3, rats were injected with LPS and administered orally with SOD (Glisodin®) 1000 IU daily. SOD was given for 16 weeks by oral gavage with a flexible feeding tube, given once daily in the morning, and then rats were intraperitoneally injected with LPS at a dose of 10 mg/kg body weight. Control group (Groups I and II) rats were given an equal volume of saline for 16 weeks by oral gavage and then were intraperitoneally injected with saline.

All rats were sacrificed after 12 h of injection of LPS, and the kidney tissue was collected, fixed in 10% formalin, and then embedded in paraffin to collect the kidney. Anesthesia with ketamine and xylazine and cervical dislocation were performed as the sacrifice methods.

### 2.3. Histopathological Analysis of the Kidney

After 12 h of injection of LPS, the kidney tissues were collected, fixed in 10% formalin, and embedded in paraffin. After dehydrating with graded alcohols for staining and sectioning into 5-*µ*m sections, the sections were stained with hematoxylin and eosin. All sections were observed under a light microscope.

Tubular injury scores are classified into four ranging from 0 to 3, as follows:

- Score 0: normal histological picture
- Score 1: edema of tubular cells, loss of brush border, nuclear condensation, <1/3 of the tubular nucleus profile is missing
- Score 2: as with score 1, 1/3–2/3 of the tubular nucleus profile is missing
- Score 3: >2/3 of the tubular nucleus profile is missing

### 2.4. Murine Sepsis Score

The MSS system involves observing seven components, namely, appearance, level of awareness, activity, response to stimuli, eyes, respiratory rate, and breathing quality. The MSSs assigned were the average of these seven components [10].  Table 1 shows a detailed score and description of each variable in the MSS.

## 3. Results and Discussion

### 3.1. Results

The results showed that the animals' weights were similar in each group (Table 2), which indicated that there was no difference in rat body weight during the study period.

MSS was used in this study to evaluate the severity of each group.  Table 3 shows that the MSSs increased significantly in the group that did not receive Glisodin®. These data suggest that Glisodin® decreases the severity of sepsis in rats.

**Figure 1:**
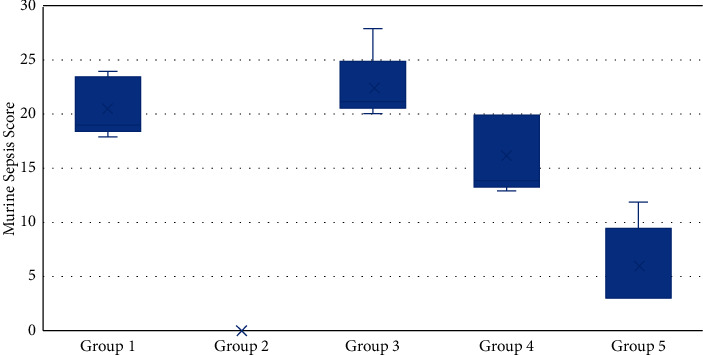
Murine sepsis score based on groups of experimental animals at the laboratory of the experimental animal at UPT, Faculty of Veterinary Medicine, Unsyiah, in 2020 (*n* = 25).

Only Group V has a symmetrical MSS distribution, as shown in Figure 1. While the other groups were asymmetrical, with the majority of median values clustered in quartile 1 (Q1). In contrast, the MSS distribution diagram is a line in Group II because there is only one value.

The MSS was also examined in sepsis rats that received SOD Glisodin compared with healthy rats (Table 4). The optimal doses of SOD Glisodin 1000 IU significantly decreased the MSS in sepsis rats. In addition, there was no significant difference in MSS in rats that received optimal doses of SOD Glisodin compared with healthy rats.

Furthermore, the highest tubular injury score was also obtained in this result (Table 5). Although there were differences between groups, a significant difference test analysis of the tubular injury score (*p* < 0.05) was obtained only between Group II and the other groups.

**Figure 2:**
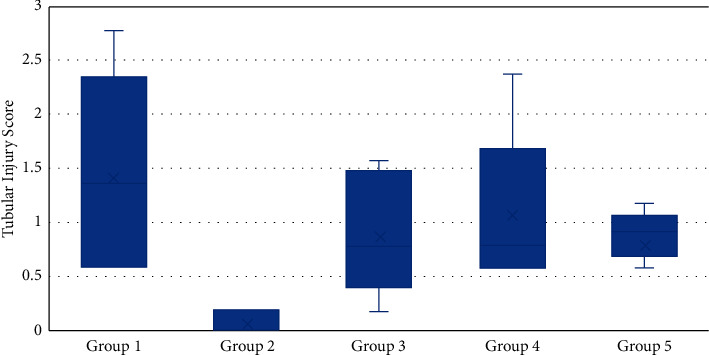
Tubular injury score based on groups of experimental animals in the laboratory of animal medicine, Faculty of Veterinary Medicine, Unsyiah, in 2020 (*n* = 25).

We also found that only Group IV shows a symmetrical distribution of the tubular injury score as shown in Figure 2. In contrast with Groups I and IV, which shows asymmetrical distribution with the median values scattered in Q3. Additionally, the rest, namely, Groups II and III, are asymmetrical, but most of the median values are scattered in Q1.

**Figure 3:**
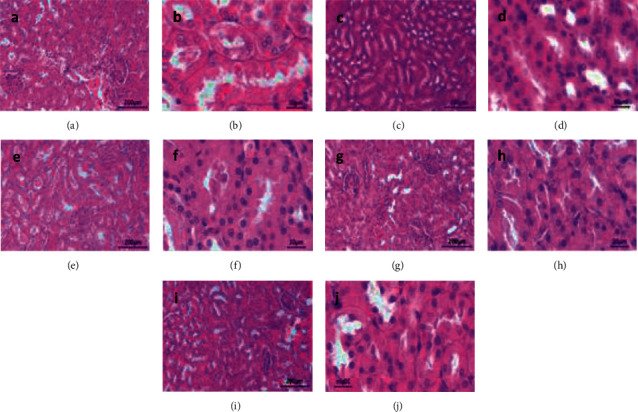
Histology of rats' kidney tissue based on the treatment group. (a) Group I at 10x magnification. (b) Group I at 40x magnification. (c) Group II at 10x magnification. (d) Group II at 40x magnification. (e) Group III at 10x magnification. (f) Group III at 40x magnification. (g) Group IV at 10x magnification. (h) Group IV at 40x magnification. (i) Group V at 10x magnification. (j) Group V at 40x magnification.

The histological features of the rat's kidney tissue are shown in Figure 3. Observations were made through an optical microscope with two magnification types, such as 10× and 40×. Meanwhile, the tubular injury score can be obtained in Table 5.

Administering SOD Glisodin has a significant effect on MSS.  Table 6 describes the contribution of SOD Glisodin to the change in MSS as 91.4%, or other factors influenced only 9.6%. The B value is obtained with the most considerable negative value in the group given SOD Glisodin of 1000 IU against the negative control group. This condition indicates that the higher dose of SOD Glisodin injection can affect the MSS value that is formed.

We can conclude that the injection of SOD Glisodin at doses of 500 and 1000 IU can be a protective factor against septic conditions in rats. The ideal dose given to rats that became experimental animals in this study is 1000 IU.

Table 7 also describes how the MSS affects the tubular injury score linearly. The higher the value of the independent variable, the higher the value of the dependent variable. The condition of sepsis in rats will affect the severity of tubular damage in the rats' kidneys by 19.8%. We can conclude that the more severe sepsis occurs, the more severe the renal tubular damage will be.

### 3.2. Discussion

Sepsis is a complex syndrome characterized by an imbalance between proinflammatory and anti-inflammatory responses to pathogens [9]. During the development of sepsis, a large number of reactive oxygen species (ROS) and nitric oxide (NO) will be produced [12]. ROS is very reactive, so it readily reacts with other compounds such as lipids, DNA, and proteins. Oxidation of ROS causes tissue or organ damage that can lead to disease [13]. Excess production of ROS and NO can cause oxidative stress due to the body's antioxidants' failure to ward off free radicals produced from ROS and NO. Antioxidants have free radical scavenging activities, which protect the body from various diseases caused by free radicals [14]. SOD is a natural antioxidant in the body that can prevent free radicals [15].

SOD is a metalloenzyme that can catalyze the release of superoxide radicals into hydrogen peroxide and oxygen. Mammals have three distinct SOD isoforms, namely, copper/zinc-SOD (Cu/ZnSOD, SOD-1), manganese-SOD (MnSOD, SOD-2), and extracellular-SOD (ECSOD, SOD-3). These SOD isoforms act as catalysts to break down free radical molecules, namely, superoxide anions, into molecular oxygen and hydrogen peroxide [16]. Damage to the respiratory system and kidney tissue is the most common form of organ dysfunction in patients with sepsis [17]. Constantino et al. (2017) [9] explained that AKI is the main well-known complication of sepsis.

One of the antioxidants that can decrease sepsis is SOD because it is the first defense enzyme found in body cells [13]. In the present study, the MSS and renal tubular damage in a rat model were used to evaluate the possible role of SOD in sepsis-induced AKI. Our results demonstrated that the SOD Glisodin treatment significantly decreased MSS and renal tubular damage.

The role of SOD in the severe sepsis phase has been described in several studies that found the imbalance of SOD levels may increase the oxidative stress during sepsis, and it can induce severe sepsis and involve organ damage [14, 18]. Oxidative stress and myeloperoxidase (MPO)–a hemoprotein released into the extracellular fluid during the inflammatory process–may be involved in organ damage in sepsis through direct and indirect mechanisms by modulation of transcription factor activation [12, 19, 20]. Furthermore, increased levels of catalase are also associated with organ damage in sepsis [21, 22]. However, efforts to explain the proper cellular and molecular interactions in sepsis continue to yield exciting information.

One of the organ damages that is feared in sepsis is AKI. The incidence of AKI in sepsis is approximately 16.2%, and the majority of these cases require renal replacement therapy [22]. The severity of AKI is highly correlated with the severity of sepsis [23], and adequate sepsis control will improve the prognosis of AKI [24]. Administration of SOD Glisodin may be an alternative therapy to control sepsis and reduces renal tubular damage, as we found in this animal model study. We believe the findings of this study can be the basis for developing the administration of SOD in cases of sepsis to prevent worsening of the prognosis. Further studies are still needed to validate these results.

## 4. Conclusions

MSS and tubular injury score were found to be decreased in the sample group added with the antioxidant SOD. These antioxidants play a role in suppressing oxidative stress that can cause AKI. SOD with a dose of 1000 IU was found to be very good at inhibiting the occurrence of AKI induced by sepsis compared to SOD with a dose of 250 and 500 IU. This shows that the antioxidant SOD can prevent the occurrence of AKI caused by sepsis by suppressing the occurrence of oxidative stress.

## Figures and Tables

**Table 1:** Murine sepsis score.

Murine sepsis score					
Variables	Score and description				
	0	1	2	3	4
Appearance	Coat is smooth	Patches of hair piloerected	Majority of the back is piloerected	Piloerection may or may not be present, and the mouse appears “puffy”	Piloerection may or may not be present, and the mouse appears emaciated
Level of consciousness	Mouse is active	The mouse is active but avoids standing upright	Mouse activity is noticeably slowed. The mouse is still ambulant	Activity is impaired. Mouse only moves when provoked, and movements have a tremor	Activity is severely impaired. Mouse remains stationary when provoked, with possible tremor
Activity	A normal amount of activity. The mouse is doing all the eating, drinking, climbing, running, and fighting	Slightly suppressed activity. The mouse is moving around the bottom of the cage	Suppressed activity. The mouse is stationary with occasional investigative movements	No activity. Mouse is stationary	No activity. Mouse is experiencing tremors, particularly in the hind legs
Response to stimulus	The mouse responds immediately to auditory stimulus or touch	Slow or no response to an auditory stimulus; strong response to touch (moves to escape)	No response to an auditory stimulus; moderate response to touch (moves a few steps)	No response to an auditory stimulus; mild response to touch (no locomotion)	No response to an auditory stimulus. Little or no response to touch. It cannot right itself if pushed over
Eyes	Open	Eyes not fully open, possibly with secretions	Eyes at least half-closed, possibly with secretions	Eyes half-closed or more, possibly with secretions	Eyes closed or milky
Respiration rate	Normal, rapid mouse respiration	Slightly decreased respiration (rate not quantifiable by eye)	Moderately reduced respiration (the rate at the upper range of quantifying by eye)	Severely reduced respiration (rate easily countable by eye, 0.5 s between breaths)	Extremely reduced respiration (>1 s between breaths)
Respiration quality	Normal	Brief periods of labored breathing	Labored, no gasping	Labored with intermittent gasps	Gasping

Source: Shrum et al. (2014) [11].

**Table 2:** Descriptive data on experimental animal weight at the laboratory of the experimental animal at UPT, Faculty of Veterinary Medicine, Unsyiah, in 2020 (*n* = 25).

No.	Weight	Group	*N*	Mean (g)	SD (g)	Min (g)	Max (g)	*p* ^*∗*^	*p* ^*∗∗*^
1	Start	I	5	231.00	17.99	216.00	259.00	0.195	0.121
		II	5	217.40	18.26	200.00	246.00	0.354	
		III	5	233.60	22.10	210.00	267.00	0.698	
		IV	5	241.80	18.21	216.00	267.00	0.769	
		V	5	248.80	14.62	227.00	262.00	0.451	
2	End	I	5	335.00	32.02	290.00	370.00	0.570	0.404
		II	5	374.20	38.46	325.00	424.00	0.823	
		III	5	322.40	75.00	257.00	443.00	0.249	
		IV	5	323.00	44.22	256.00	360.00	0.302	
		V	5	336.60	22.61	307.00	368.00	0.996	

^*∗*^Data normality test with the Shapiro–Wilk test. ^*∗∗*^ANOVA test.

**Table 3:** Descriptive data of murine sepsis scores on experimental animals at the laboratory of experimental animal at UPT, Faculty of Veterinary Medicine, Unsyiah, in 2020 (*n* = 25).

No.	Group	n	Mean	SD	Min	Max	*p* ^*∗*^	*p* ^*∗∗*^
1	I	5	20.60	2.70	18.00	24.00	0.166	0.001
2	II	5	0	0	0	0	–	
3	III	5	22.40	3.21	20.00	28.00	0.030	
4	IV	5	16.20	3.49	13.00	20.00	0.039	
5	V	5	6.00	3.74	3.00	12.00	0.235	

^*∗*^Data normality test with the Shapiro–Wilk test. ^*∗∗*^Kruskal–Wallis test.

**Table 4:** Comparison of murine sepsis scores of experimental animals in Group I compared to Groups III, IV, V, and II in the laboratory of experimental animal at UPT, Faculty of Veterinary Medicine, Unsyiah, in 2020 (*n* = 25).

No.	Group	Group	Mean difference	SE	*p* ^*∗*^
1	I	III	−1.80	1.88	0.990
2		IV	4.40	1.97	0.453
3		V	14.60	2.06	0.002
4		II	20.60	1.21	0.001

^*∗*^Post hoc Tamhane test.

**Table 5:** Descriptive data of tubular injury scores in experimental animals at the laboratory of experimental animal at UPT, Faculty of Veterinary Medicine, Unsyiah, in 2020 (*n* = 25).

No.	Group	n	Mean	SD	Min	Max	*p* ^*∗*^	*p* ^*∗∗*^
1	I	5	1.44	0.92	0.60	2.80	0.437	0.018
2	II	5	0.08	0.11	0	0.20	0.006	
3	III	5	0.92	0.58	0.20	1.60	0.742	
4	IV	5	1.08	0.76	0.60	2.40	0.019	
5	V	5	0.92	0.23	0.60	1.20	0.814	

^*∗*^Data normality test with the Shapiro–Wilk test. ^*∗∗*^Kruskal–Wallis test.

**Table 6:** Results of the analysis of the effect of administering SOD Glisodin on murine sepsis scores based on the treatment group of experimental animals at the laboratory of animal medicine at UPT, Faculty of Veterinary Medicine, Unsyiah, in 2020 (*n* = 25).

No.	Independent variable	Dependent variable	*p* ^*∗*^	*R* ^2^	Group	B	*p* ^*∗∗*^
1	SOD Glisodin	Murine sepsis score	0.001	0.914	II	−20.600	0.001
					III	1.800	0.348
					IV	−4.400	0.029
					V	−14.600	0.001
					I	0^a^	–

^a^This parameter is set to zero because it is redundant. ^*∗*^Tests of between-subjects effects. ^*∗∗*^Parameter estimates.

**Table 7:** Results of analysis of the effect of murine sepsis score on tubular injury score in experimental animals at the laboratory of experimental animal at UPT, Faculty of Veterinary Medicine, Unsyiah, in 2020 (*n* = 25).

No.	Independent variable	Dependent variable	*B*	*p*	*R* ^2^
1	Murine sepsis score	Tubular injury score	0.034	0.026	0.198

## Data Availability

Data regarding this study are available from the corresponding author upon reasonable request.

## References

[1] Torio C. M., Andrews R. M. (2011). *National Inpatient Hospital Costs: The Most Expensive Conditions by payer*.

[2] Seymour C. W., Kerti S. J., Lewis A. J. (2019). Murine sepsis phenotypes and differential treatment effects in a randomized trial of prompt antibiotics and fluids. *Critical Care*.

[3] Kolhe N. V., Stevens P. E., Crowe A. V. (2008). Case mix, outcome and activity for patients with severe acute kidney injury during the first 24 hours after admission to an adult, general critical care unit: application of predictive models from a secondary analysis of the ICNARC Case Mix Programme database. *Critical Care (London, England)*.

[4] Nguyen M. T., Devarajan P. (2008). Biomarkers for the early detection of acute kidney injury. *Pediatric Nephrology*.

[5] Surachtono S. (2011). Acute kidney injury on sepsis. *The Indonesian Journal of Anesthesiology and Critical Care*.

[6] Teixeira-da-Cunha M. G. A., Gomes R. N., Roehrs N. (2013). Bacterial clearance is improved in septic mice by platelet-activating factor-acetylhydrolase (PAF-AH) administration. *PLoS One*.

[7] Tupchong K., Koyfman A., Foran M. (2015). Sepsis, severe sepsis, and septic shock: a review of the literature. *African Journal of Emergency Medicine*.

[8] Aksu U., Demirci C., Ince C. (2011). The pathogenesis of acute kidney injury and the toxic triangle of oxygen, reactive oxygen species and nitric oxide. *Contributions to Nephrology*.

[9] Constantino L., Galant L. S., Vuolo F. (2017). Extracellular superoxide dismutase is necessary to maintain renal blood flow during sepsis development. *Intensive Care Medicine Experimental*.

[10] Mai S., Sharma N., Kwong A. C. (2018). Body temperature and mouse scoring systems as surrogate markers of death in cecal ligation and puncture sepsis. *Intensive care medicine experimental*.

[11] Shrum B., Anantha R. V., Xu S. X. (2014). Aroburst scoring system to evaluate sepsis severity in an animal model. *BMC Research*.

[12] Macdonald J. (2003). Oxidative stress and gene expression in sepsis. *British Journal of Anaesthesia*.

[13] Prasetya R. J., Zainumi C. M. (2019). Role of superoxide dismutase (SOD) gene ala16val in sepsis. *International Journal of Innovative Science and Research Technology*.

[14] Kumar S., Gupta E., Kaushik S. (2018). Evaluation of oxidative stress and antioxidant status: correlation with the severity of sepsis. *Scandinavian Journal of Immunology*.

[15] Warner B. W. (1987). Superoxide dismutase in rats with sepsis. *Archives of Surgery*.

[16] Sciskalska M., Oldakwoska M., Marek G. (2020). Changes in the activity and concentration of superoxide dismutase isoenzymes (Cu/Zn SOD, MnSOD) in the blood of healthy subjects and patients with acute pancreatitis. *Antioxidants*.

[17] Schrier R. W., Wang W. (2004). Acute renal failure and sepsis. *New England Journal of Medicine*.

[18] Andrades M., Ritter C., Moreira J. C. F. (2005). Oxidative parameters difference during non-lethal and lethal ssepsis development. *Journal of Surgical Research*.

[19] Andrades M., Ritter C., de Oliviera M. R. (2011). Antioxidant treatment reverses organ failure in rat model of sepsis: role of antioxidant enzymes imbalance, neutrophil infiltration, and oxidative stress. *Journal of Surgical Research*.

[20] Linawati S. (2012). *Perbandingan Marker Inflamasi Antara Sindroma Koroner Akut Dan Non Sindroma Koroner Akut: Myeloperoxidase Dan High Sensitive C-Reactive Protein Sebagai Marker Inflamasi Pada dislipidemia*.

[21] Baumgart K., Simkova V., Wagner F. (2009). Effect of SOD-1 over-expression on myocardial function during resuscitated murine septic shock. *Intensive Care Medicine*.

[22] Uchino S., Kellum J. A., Bellomo R. (2005). Acute renal failure in critically ill patients: a multinational, multicenter study. *Journal of the American Medical Association*.

[23] Setiawan D., Harun H., Azmi S. (2016). Biomarker acute kidney injury (AKI) pada sepsis. *Jurnal Kesehatan Andalas*.

[24] Kairupan J. D., Palar S. (2020). Gangguan ginjal akut et kausa sepsis: laporan kasus. *Medical Scope Journal*.

